# Three-dimensional morphological study of the proximal femur in Crowe type IV developmental dysplasia of the hip

**DOI:** 10.1186/s13018-021-02789-5

**Published:** 2021-10-18

**Authors:** Yuhui Yang, Weihong Liao, Weiqun Yi, Hai Jiang, Guangtao Fu, Yuanchen Ma, Qiujian Zheng

**Affiliations:** 1 Department of Orthopedics, Guangdong Provincial People’s Hospital, Guangdong Academy of Medical Sciences, 106, Zhongshan Second Road, Yuexiu District, Guangzhou, 510080 Guangdong Province People’s Republic of China; 2 Department of Operating Room, Guangdong Provincial People’s Hospital, Guangdong Academy of Medical Sciences, 106, Zhongshan Second Road, Yuexiu District, Guangzhou, 510080 People’s Republic of China

**Keywords:** Developmental dysplasia of the hip, Crowe IV, Femoral morphology, Three-dimensional reconstruction, Total hip arthroplasty

## Abstract

**Background:**

When performing femoral reconstruction in patients with Crowe type IV developmental dysplasia of the hip (DDH), anatomical deformity presents many technical challenges to orthopedic surgeons. The false acetabulum is suggested to influence load transmission and femoral development. The aim of this study was to describe the morphological features of dysplastic femurs in Crowe type IV DDH and further evaluate the potential effect of the false acetabulum on morphological features and medullary canal of Crowe type IV femurs.

**Methods:**

We analyzed preoperative computed tomography scans from 45 patients with 51 hips (25 hips without false acetabulum in the IVa group and 26 hips with false acetabulum in the IVb group) who were diagnosed with Crowe type IV DDH and 30 normal hips in our hospital between January 2009 and January 2019. Three-dimensional reconstruction was performed using Mimics software, and the coronal femoral plane was determined to evaluate the following parameters: dislocation height, dislocation ratio, height of the femoral head (FH), height of the greater trochanter (GT), GT–FH height discrepancy, height of the isthmus, neck-shaft angle, femoral offset and anteversion of the femoral neck. The mediolateral (ML) width, anterolateral (AP) width and diameter of medullary canal of the proximal femur were measured on the axial sections. Further, canal flare index (CFI), metaphyseal-CFI and diaphyseal-CFI were also calculated.

**Results:**

Compared with the normal femurs, the Crowe type IV DDH femurs had a higher femoral head, larger GT–FH height discrepancy, larger femoral neck anteversion, higher isthmus position and smaller femoral offset. Dislocation height and dislocation rate were significantly larger in the IVa DDH group (65.34 ± 9.83 mm vs. 52.24 ± 11.42 mm). Further, the IVb femurs had a significantly lower isthmus position, larger neck-shaft angle and smaller femoral neck anteversion than IVa femurs. The ML, AP canal widths and the diameter of medullary canal in both DDH groups were significantly smaller than the normal group. Dimensional parameters of IVa femurs were also narrower than IVb femurs in most sections, but with no difference at the level of isthmus. According to the CFIs, the variation of proximal medullary canal in IVb femurs was mainly located in the diaphyseal region, while that in IVa femurs was located in the whole proximal femur.

**Conclusions:**

High dislocated femurs are associated with more anteverted femoral neck, smaller femoral offset and narrower medullary canal. Without stimulation of the false acetabulum, IVa DDH femurs were associated with higher dislocation and notably narrower medullary canal, whose variation of medullary canal was located in the whole proximal femur.

## Introduction

Developmental dysplasia of the hip (DDH) has been widely considered to be one of the most common causes of premature osteoarthritis and represents a spectrum of pathological abnormalities in the acetabular and femoral structures [1–3]. Total hip arthroplasty (THA) is considered to be an effective and standard treatment for patients with end-stage hip diseases. However, the severe deformity of hip dysplasia presents many technical challenges to orthopedic surgeons [4, 5]. Compared with osteoarthritis, the increased femoral complications of THA in DDH patients are associated with pathoanatomical features of the femoral medullary canal, especially in high dislocated Crowe type IV DDH patients [6–8].

Due to the presence of a false acetabulum, the Crowe type IV DDH hips were divided into IVa (without false acetabulum) and IVb (with false acetabulum) [9, 10]. Zhou et al. [11] suggested that the presence of a false acetabulum is an important factor for determining dislocation height and subtrochanteric osteotomy application in THA. Hartofilakidis et al. [6, 12] revealed that long-term implant survival after THA was significantly worse in the high dislocation DDH patients without false acetabulum, which may be a result of the anatomical conditions in two subtypes. The presence of false acetabulum in high dislocation hip was suggested to play an important role in femoral development. The Dorr type of femoral medullary canal was also proved to be associated with periprosthetic femoral fracture in patients with DDH [7]. Thus, the morphological features of the femoral proximal medullary canal are of major concern for improving preoperative planning and avoiding perioperative complications in Crowe type IV DDH patients.

Compared with conventional 2-dimensional (2D) radiographs, 3-dimensional (3D) images are more effective in detecting the morphological features and in assessing the medullary shape of the dysplastic femurs [4, 13]. To our knowledge, few studies have addressed the morphology and proximal femoral anatomy of the dysplastic femur in type IV DDH. We hypothesize that there exist morphological abnormalities in Crowe type IV femurs, and the presence of false acetabulum can also influence femoral development and anatomic structures. In this study, we sought to compare (1) the 3D morphological features between normal and dysplastic femurs in Crowe type IV DDH and (2) the 3D femoral anatomy and medullary canal variation between IVa and IVb DDH femurs.

## Patients and methods

### Study subjects

**Fig. 1:**
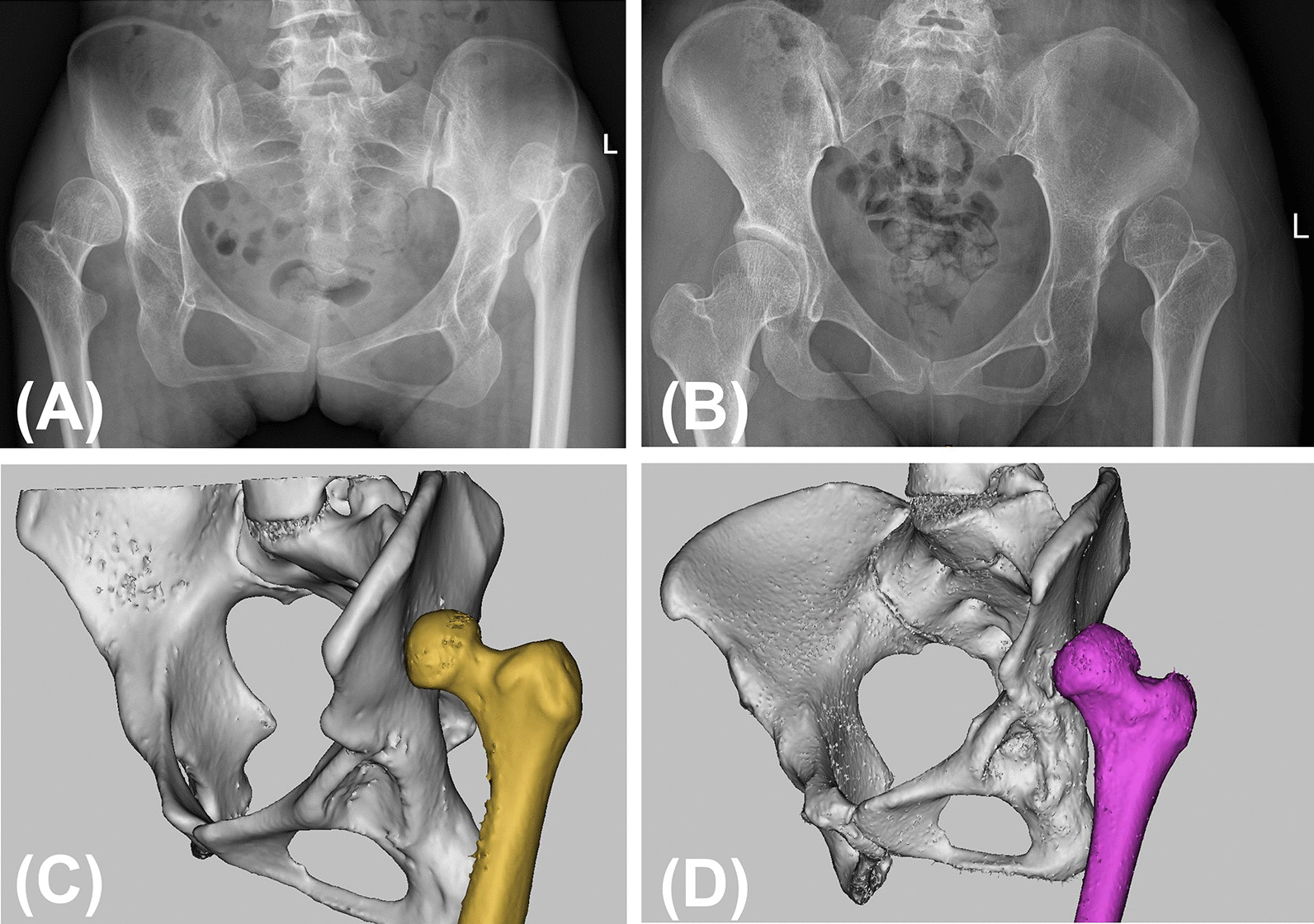
AP radiograph and 3D model two types of Crowe IV DDH. **A**, **C** A 22-year-old female patient in Crowe type IVa group (L: IVa, R: III). **B**, **D** A 30-year-old female patient in Crowe type IVb group (L: IVb)

The study was approved by the institutional review board of Guangdong Provincial People’s Hospital (IRB: 2019528HR1). We retrospectively reviewed the preoperative imaging data of 352 patients (496 hips) with DDH between January 2009 and January 2019. According to the Crowe classification, 52 patients (60 hips) were graded as type IV DDH on standing anteroposterior pelvic radiographs. The height of the dislocation was measured on standard AP plain radiographs and was defined as the vertical distance between the head-neck junction and the line connecting the lower edges of the bilateral teardrops. The exclusion criteria included (1) patients who underwent prior hip surgery; (2) patients with a dislocation attributed to infection or trauma; and (3) patients who did not have preoperative CT data or had substandard CT data. Thus, 51 dysplastic hips in 45 patients met the inclusion criteria and were retrospectively evaluated. The included patients were further divided as follows: group IVa, a dislocated femoral head located within the abductor muscle mass, and group IVb, evidence of the formation of a false acetabulum (Fig. 1). Herein, 25 hips were included in the IVa group and 26 hips were included in IVb group, with 3 patients affected by bilateral Crowe type IV DDH in each group. Fifteen patients (30 hips) without hip disease or deformities who had undergone computed tomography angiography (CTA) to diagnose vascular diseases were chosen as controls. Demographic data for the subjects are shown in Table 1.

**Table 1:** Demographic data

	DDH (*n* = 51)		*p*	Normal (*n* = 30)
	IVa (*n* = 25, without false acetabulum)	IVb (*n* = 26, with false acetabulum)		
Patient (No.)	22	23	–	15
Gender(F/M)	2/20	3/20	0.673	2/13
Age (y)	41.91 ± 15.27 (21–70)	43.70 ± 11.52 (23–65)	0.630	41.40 ± 8.04 (29–54)
Height (cm)	159.32 ± 11.81 (141–179)	156.78 ± 8.65 (143–175)	0.389	163.20 ± 8.04 (155–185)
Weight (kg)	60.36 ± 10.69 (40–80)	54.91 ± 12.29 (30–84)^#^	0.317	65.07 ± 8.55 (49–87)
BMI (kg/m^2^)	23.84 ± 3.91 (18.73–30.08)	22.43 ± 5.25 (13.33–35.42)	0.277	24.47 ± 2.97 (18.00–29.14)

Values are expressed as the mean and the standard deviation, with range in parentheses; *p* means the differences between the cortical widths of type IVa DDH and type IVb DDH; ^#^*p* < 0.05, when compared with control group

### Image analysis and measurements

**Fig. 2:**
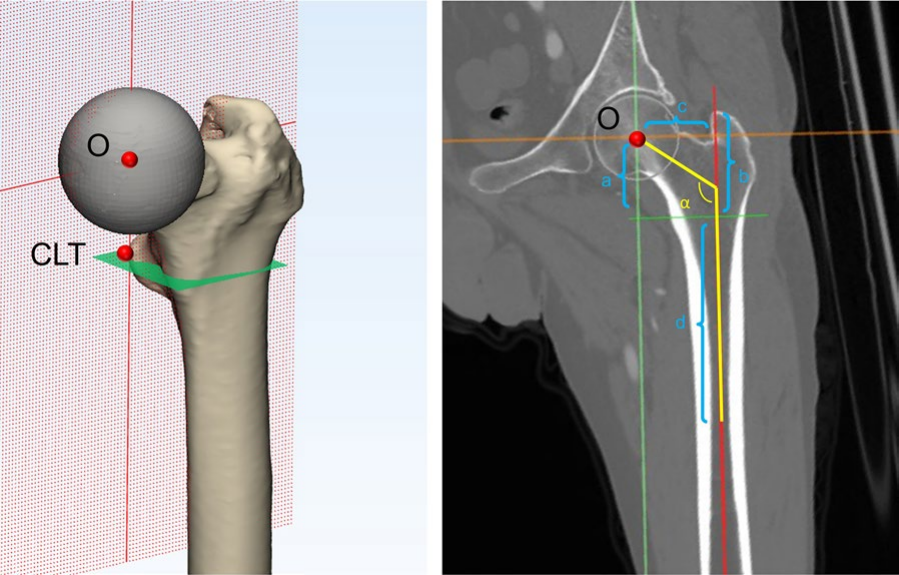
3D model and the coronal femoral plane of the proximal femur of DDH. O: femoral head center; CLT: center of the lesser trochanter; a: height of FH; b: height of GT; c: femoral offset; d: height of isthmus; ɑ: neck-shaft angle; GT = greater trochanter, FH = femoral head

Pelvic CT was performed with a Toshiba brand Aquilion CT scanner (120 kVp, 320 mA, 512 × 512 matrix; slice thickness 0.5 mm) at the Guangdong Provincial People’s Hospital. The patients were placed in a neutral supine position with the patellae facing the ceiling. Scanning was performed from the iliac crest to the complete femoral condyles. All standard CT slices were saved in digital imaging and communications in medicine (DICOM) format and imported into Mimics 19.0 software (Materialise, Leuven, Belgium) for 3D reconstruction. Accordingly, the 3D, coronal, sagittal and transverse views were presented simultaneously in Mimics software. First, the center of the femoral head was established based on the fitting sphere of femoral head. Second, the center of the lesser trochanter (CLT) is the most prominent point on the lesser trochanter on the 3D model. The central axis of the femoral medullary canal was defined as the line between the geometric centers of the medullary canal at the level of 20 mm below the CLT and isthmus of the femur. Thus, the coronal femoral plane was determined by the femoral head center and the central axis of the femoral medullary (Fig. 2A).

Based on the coronal plane, the 3D model and CT images were reoriented and the following morphological evaluations included (Fig. 2B): (1) Height of the femoral head (FH): the vertical distance between the femoral head center and level of CLT. (2) Height of the greater trochanter (GT): the vertical distance between the tip of the greater trochanter and the level of CLT. (3) GT–FH height discrepancy. (4) Height of the isthmus: the vertical distance between the level of isthmus and CLT. (5) Neck-shaft angle: the femoral neck axis was first defined by connecting the femoral head center and the midpoint of the narrowest part of the femoral neck; the neck-shaft angle was defined as the angle between the femoral neck axis and the central axis of the femoral medullary canal. (6) Femoral offset: the horizontal distance between the femoral head center and the central axis of the femoral medullary canal. (7) Anteversion of the femoral neck: the angle between the post-condylar line and the projection of the femoral neck axis on the axial plane.

**Fig. 3:**
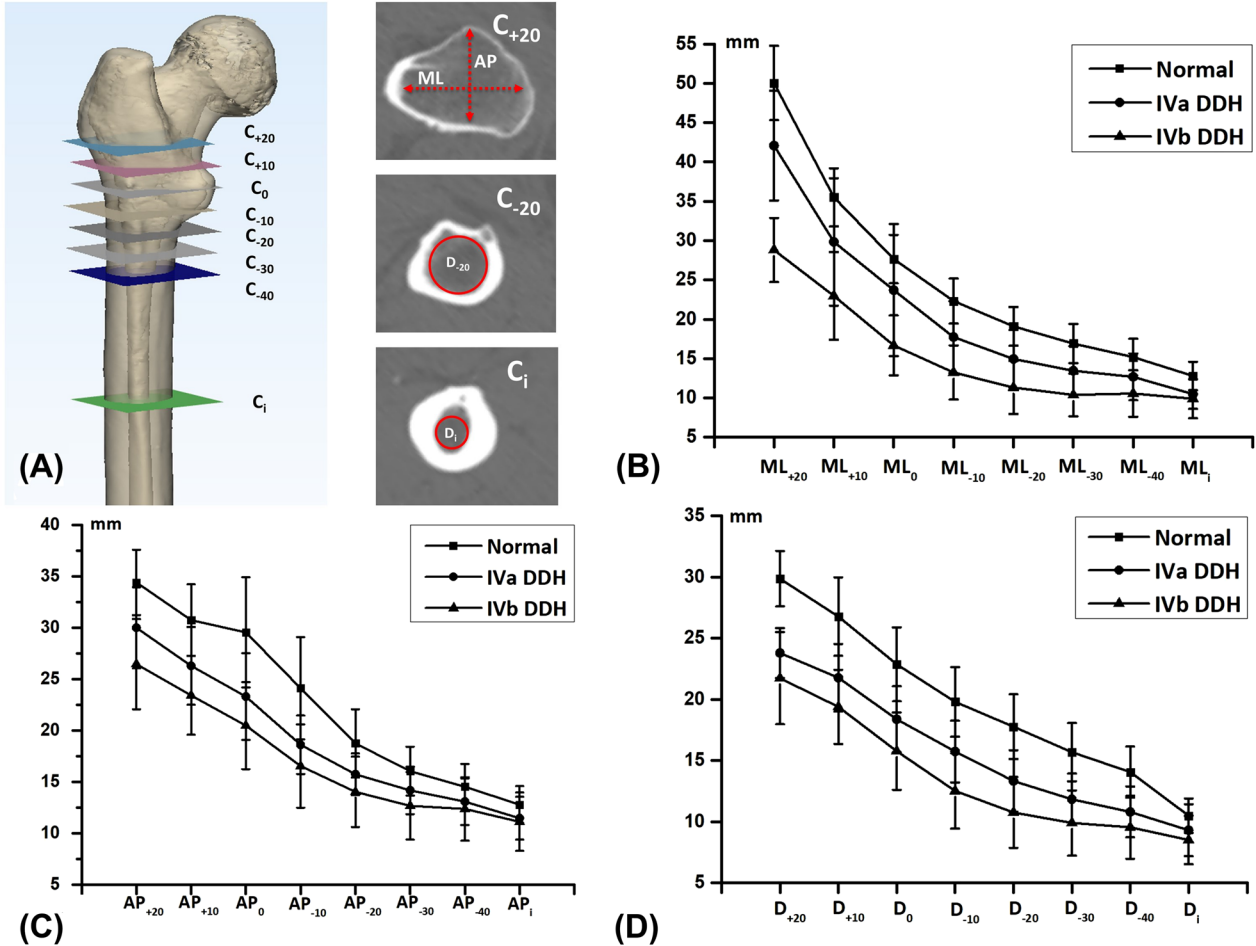
Measurements of medullary canal dimensional parameters. **A** Definition of the femoral medullary canal axial sections at 1-cm intervals from 2 cm above the CLT (C_+20_) to 4 cm below the CLT (C_−40_) and the section of isthmus (C_i_). Dimensional parameters contain ML canal width, AP canal width and diameter of the best-fitting circle of the medullary canal. **B** ML canal widths in IVa, IVb and normal groups. **C** AP canal widths in IVa, IVb and normal groups. **D** Diameters in IVa, IVb and normal groups

Refer to the medullary canal parameters, femoral model was evaluated from the level of 20 mm above the CLT (C_+20_) to the level of 40 mm below the CLT (C_−40_) at 10-mm intervals (Fig. 3A). Based on the method described by Noble and Sugano et al. [14, 15], the mediolateral (ML) and anteroposterior (AP) widths of medullary canal were measured. Accordingly, the ML width was measured in planes parallel to the femoral neck axis and the AP width was measured perpendicular to the ML width. Further, a circle was also created to best fill the inner contour of medullary canal [4]. The ML width, AP width and fitting circle diameter of medullary canal were measured to describe the medullary morphology on each axial plane, as well as the level of isthmus. As described previously, canal flare index (CFI) was calculated as the ratio between the widths of the medullary canal at the level of the 20 mm above the CLT and the isthmus [16]. Metaphyseal canal flare index (MCFI) was calculated as the ratio between the widths of the medullary canal at the levels of 20 mm above and 20 mm below the CLT [17]. Diaphyseal canal flare index (DCFI) was calculated as the ratio between the widths of the medullary canal at the level of 20 mm below the CLT and the isthmus [4].

### Statistical analysis

Kolmogorov–Smirnov tests were performed to determine the distributions of the data. For parametric data, when the variances in groups were same, one-way ANOVA was used to compare the differences among groups, followed by the least significant difference (LSD) method for pairwise comparisons. For nonparametric data or when variances in groups were different, Kruskal–Wallis analysis of variance was performed, followed by Dunn’s test for pairwise comparisons. For assessing interobserver reliability, two analysts performed point selection and corresponding measurements independently. For assessing intraobserver reliability, measurements were repeated one month later by the same analysts. The intraclass correlation coefficient was used to calculate interobserver and intraobserver effects. A post hoc power calculation was determined by the statistical power analyses G Power 3.1 to eliminate type II error [18]. All statistical analyses were performed using SPSS version 21.0 (SPSS, Chicago, IL, USA), and a *p* value < 0.05 was considered statistically significant.

## Results

The anatomy of the dysplastic femur and corresponding bony structures were evaluated following 3D reconstruction. As listed in Table 1, patients in IVa, IVb and normal groups had a comparable mean age and body mass index (BMI). Intraclass correlation coefficient results of the intraobserver and interobserver reliabilities for all the measurement indices, evaluated by the one-way random-effects model, ranged from 0.93 to 0.97 and from 0.82 to 0.93, respectively. Post hoc power analysis showed a power > 0.84 for detecting a significant difference.

According to the measurements of standard AP plain radiographs, both the dislocation height and dislocation rate were significantly higher in the IVa DDH group (65.34 ± 9.83 mm) than in the IVb DDH group (52.24 ± 11.42 mm). Compared with the normal femurs, the dislocated femurs had a higher femoral head, larger GT–FH height discrepancy between, larger femoral neck anteversion and smaller femoral offset. Moreover, the position of the femoral isthmus was significantly higher in the DDH groups. Further, the IVb DDH had a significantly lower isthmus position, larger neck-shaft angle and smaller femoral neck anteversion than that in IVa DDH. There was no significant difference in the position of FH and GT and femoral offset between DDH groups (Table 2).

**Table 2:** Parameters of proximal femoral anatomy for DDH and control groups

	DDH (*n* = 51)		*p*	Normal (*n* = 30)
	IVa (*n* = 25, without false acetabulum)	IVb (*n* = 26, with false acetabulum)		
Height of the dislocation (mm)	65.34 ± 9.83 (46.79–81.35)	52.24 ± 11.42 (39.18–76.91)	0.000**	/
Dislocation rate (%)	31.88 ± 4.10 (24.52–38.62)	24.99 ± 4.81 (20.08–36.30)	0.000**	/
Height of the FH (mm)	38.99 ± 7.81 (23.12–53.19)^##^	37.35 ± 6.48 (19.80–47.41)^##^	0.395	45.55 ± 6.25 (34.53–64.37)
Height of the GT (mm)	54.72 ± 4.90 (46.27–64.24)	51.87 ± 4.72 (44.57–61.91)^##^	0.052	55.88 ± 5.71 (47.08–71.13)
GT–FH height discrepancy (mm)	15.73 ± 5.13 (6.90–29.69)^##^	14.52 ± 5.42 (1.92–27.65)^##^	0.356	10.34 ± 3.37 (4.25–16.81)
Height of the isthmus (mm)	68.16 ± 26.87 (25.18–128.02)^##^	87.93 ± 23.71 (35.63–125.23)^##^	0.023*	114.10 ± 15.82 (82.28–147.91)
Femoral offset (mm)	31.41 ± 7.27 (16.51–46.80)^##^	33.90 ± 3.72 (27.02–42.29)^##^	0.345	41.38 ± 3.28 (34.78–49.40)
Neck-shaft angle (°)	114.64 ± 7.58 (97.79–127.65)^##^	126.51 ± 7.58 (113.62–143.87)	0.000**	125.44 ± 4.72 (117.99–136.16)
Femoral neck anteversion (°)	41.35 ± 14.55 (3.26–65.19)^##^	25.94 ± 11.06 (4.13–51.63)^##^	0.000**	14.96 ± 9.03 (2.40–35.84)

Values are presented as the mean and the standard deviation, with range in parentheses; *p* means the differences between the cortical widths of type IVa DDH and type IVb DDH; *p < 0.05, **p < 0.01 for the comparison between the DDH groups; ^#^*p* < 0.05, ^##^*p* < 0.01, when compared with control group

*GT* greater trochanter, *FH* femoral head

Regarding the morphology of medullary canal (Fig. 3B), the ML, AP canal widths and the diameter of medullary canal in both DDH groups were significantly smaller than normal group from C_+20_ to C_−40_ and at the level of isthmus. Within DDH groups, the AP width and diameter of the IVa femurs were smaller than the IVb femurs from C_20_ to C_−30_, similar to ML width from C_+20_ to C_−40_, while there was no significant difference in anatomic measurements at the level of isthmus between IVa and IVb groups (Table 3). As detailed in Table 4, ML-CFI and D_+20_/Di of DDH femurs were smaller than normal femurs. Further, IVa femurs appeared to be more stovepipe-like than IVb femurs in the ML dimension (3.02 ± 0.53 mm vs. 3.99 ± 0.71 mm, *p* = 0.000). However, there was no difference of D_+20_/Di between IVa and IVb femurs. According to the metaphyseal canal, there was no statistical difference of MCFI between DDH and normal femurs in both ML and AP dimensions. While IVa femurs had a larger D_+20_/D_−20_ than IVb and normal femurs. According to the diaphyseal canal, all the quantitative ratios of DDH femurs were smaller than normal femurs, except for IVb femurs in the AP dimension. Moreover, ML-DCFI, AP-DCFI and D_−20_/Di of IVb femurs were significantly larger than IVa femurs.

**Table 3:** Parameters of medullary canal for DDH and control groups

	ML width				AP width				*D*			
	IVa	IVb	*p*	Normal	IVa	IVb	*p*	Normal	IVa	IVb	*p*	Normal
C_+20_	28.81 ± 4.07^##^	42.09 ± 6.99^#^	0.000**	50.05 ± 4.73	26.46 ± 4.38^##^	29.99 ± 3.95^##^	0.001**	34.40 ± 3.18	21.73 ± 3.76^##^	23.79 ± 2.05^##^	0.01**	29.87 ± 2.27
C_+10_	22.97 ± 5.58^##^	29.84 ± 8.1^##^	0.000**	35.51 ± 3.70	23.42 ± 3.82^##^	26.29 ± 3.78^##^	0.007**	30.74 ± 3.46	19.39 ± 3.03^##^	21.77 ± 2.77^##^	0.006**	26.77 ± 3.21
C_0_	16.68 ± 3.80^##^	23.71 ± 8.39^##^	0.000**	27.66 ± 3.08	20.48 ± 4.23^##^	23.30 ± 4.22^##^	0.034*	29.54 ± 5.35	15.77 ± 3.16^##^	18.38 ± 2.69^##^	0.002**	22.87 ± 3.02
C_−10_	13.23 ± 3.44^##^	17.73 ± 4.57^##^	0.000**	22.32 ± 2.86	16.55 ± 4.05^##^	18.62 ± 2.84^##^	0.117	24.11 ± 4.98	12.52 ± 3.08^##^	15.74 ± 2.52^##^	0.000**	19.80 ± 2.85
C_−20_	11.33 ± 3.37^##^	14.95 ± 3.70^##^	0.002**	19.10 ± 2.46	14.04 ± 3.42^##^	15.75 ± 2.02^##^	0.046*	18.75 ± 3.32	10.76 ± 2.89^##^	13.33 ± 2.52^##^	0.001**	17.77 ± 2.64
C_−30_	10.38 ± 2.70^##^	13.46 ± 3.10^##^	0.000**	16.91 ± 2.48	12.68 ± 3.28^##^	14.18 ± 2.30^#^	0.049*	16.05 ± 2.38	9.90 ± 2.67^##^	11.83 ± 2.11^##^	0.005**	15.69 ± 2.38
C_−40_	10.56 ± 2.96^##^	12.67 ± 2.97^##^	0.008**	15.19 ± 2.33	12.38 ± 3.09^##^	13.09 ± 2.27^#^	0.324	14.53 ± 2.23	9.55 ± 2.57^##^	10.80 ± 2.08^##^	0.051	14.06 ± 2.08
C_i_	9.89 ± 2.48^##^	10.48 ± 1.89^##^	0.724	12.79 ± 1.80	11.13 ± 2.84^##^	11.47 ± 2.08^#^	0.598	12.79 ± 1.80	8.51 ± 1.98^##^	9.31 ± 2.12^#^	0.126	10.5 ± 1.41

Values are presented as the mean and the standard deviation; *p* means the differences between type IVa and type IVb DDH; *p < 0.05, **p < 0.01 for the comparison between the DDH groups; ^#^*p* < 0.05, ^##^*p* < 0.01, when compared with control group

*ML width* mediolateral width, *AP width* anteroposterior width, *D* diameter of best-fitting circle

**Table 4:** Canal flare indices for DDH and control groups

	DDH (*n* = 51)		*p*	Normal (*n* = 30)
	IVa (*n* = 25)	IVb (*n* = 26)		
ML-CFI	3.02 ± 0.53^##^	3.99 ± 0.71^#^	0.000**	4.39 ± 0.40
AP-CFI	2.47 ± 0.54	2.74 ± 0.65	0.079	2.70 ± 0.40
D_+20_/Di	2.61 ± 0.43^#^	2.53 ± 0.47^##^	0.500	2.89 ± 0.41
ML-MCFI	2.68 ± 0.56	2.86 ± 0.55	0.599	2.65 ± 0.31
AP-MCFI	1.96 ± 0.45	1.96 ± 0.33	0.999	1.86 ± 0.27
D_+20_/D_−20_	2.10 ± 0.42^#^	1.83 ± 0.28	0.014*	1.82 ± 0.43
ML-DCFI	1.14 ± 0.13^##^	1.42 ± 0.22^##^	0.000**	1.68 ± 0.21
AP-DCFI	1.27 ± 0.18^##^	1.39 ± 0.18	0.037*	1.47 ± 0.23
D_−20_/Di	1.26 ± 0.16^##^	1.42 ± 0.20^##^	0.005**	1.70 ± 0.21

Values are presented as the mean and the standard deviation; *p* means the differences between type IVa and type IVb DDH; *p < 0.05, **p < 0.01 for the comparison between the DDH groups; ^#^*p* < 0.05, ^##^*p* < 0.01, when compared with control group

*CFI* canal flare index, *MCFI* metaphyseal canal flare index, *DCFI* diaphyseal canal flare index

## Discussion

Although femoral reconstruction in patients with Crowe IV DDH shows reliable advantages and clinical outcomes, the complications including intraoperative fracture [19, 20] and nonunion at the osteotomy site [21–23] still rate from 5 to 25%. The anatomical deformities presented in the proximal femur in patients with high dislocation are recognized as major surgical challenges [24, 25]. Therefore, in the present study, we evaluated the 3D-CT-based morphological analysis of proximal femoral anatomy and medullary canal in hips affected by Crowe IV DDH. According to the absence or presence of false acetabulum, we also found a series of anatomical distinctions between IVa and IVb femurs.

Commonly, the dysplastic femur was described in terms of greater anteversion, larger valgus deformity and narrower intramedullary canal [14, 26]. However, we found neck-shaft angle in the Crowe IVa group was significantly smaller than that of IVb and normal group, whereas no difference was found between IVb and normal groups. Our results were consistent with the conclusion of Liu [4] and Wang et al. [27], but Du reported that the angle in IVb was larger than the contralateral normal femur [10]. The medial offset in both DDH groups was smaller than the normal group and no difference was found between DDH groups, which was consistent with results of Du [10] and Noble et al. [15]. Similarly, we found both DDH femurs had a smaller distance between lesser trochanter and isthmus than normal femurs [4]. Regarding the femoral neck anteversion, dysplastic femurs had a significantly larger anteversion than the normal femurs. Without the restriction of false acetabulum, the IVa femurs had a notably larger anteversion than IVb femurs, which was different from the comparable results previously [27]. Compared with the normal femur, both IVa and IVb femurs had smaller head height and IVb femurs had larger greater trochanter height, resulting in larger GT–FH height discrepancy in both DDH groups. By contrast, there was no difference in the height of GT and FH between the DDH groups.

From the observations in this study, false acetabulum plays an important role in load transmission and bone remodeling. First, the presence of false acetabulum restrained the excessive dislocation of the affected femur, resulting in significantly smaller dislocation height in IVb group. Second, the isthmus position was more proximal in IVa than IVb femurs.

Together with the excessively narrow medullary canal, surgeons should pay close attention to avoid periprosthetic femoral fractures during femoral reconstruction. Third, IVa femurs had a significantly smaller neck-shaft angle and larger neck anteversion. It has been reported that the load transfer during the developing period was associated with a longer femoral neck and higher neck-shaft angle [28]. Thus, the abnormal morphology in IV femurs potentially compromising the component fixation and THA results should be adequately considered preoperatively.

Regarding the internal dimensions of the femoral canal, Argenson [3, 5] and Noble et al. [15] have conducted a series of CT-based studies and revealed the parameters were comparable for all groups of dysplastic hips and smaller than those of the control group. However, there were only a small number of patients with high dislocation, and they did not perform the subgroup analysis. In the present study, the ML, AP widths and diameter in both DDH groups were significantly smaller than those of the control group from C_+20_ to C_−40_ and at the level of isthmus. Meanwhile, the medullary canal of the Crowe IVa was narrower than IVb in almost all the sections, except for the isthmus level. Du et al. [10] also reported similar results that there was no significant dimensional difference at the level of isthmus. However, Wang et al. [27] demonstrated that the dimensional widths of femurs without false acetabulum were narrower in all the sections. In normal femurs, the ML width of proximal femoral canal is always longer than the AP width [17]. However, the ML width of IVb femurs became smaller than AP width from the C_−10_ level, and the abnormal condition of IVa femurs began from the C_+10_ level. Similar as reported previously [29–31], the abnormal bony torsion existed through the entire lower limb in dysplastic hips.

In the setting of high dislocation hip, the S-ROM cementless femoral stem (DePuy, Warsaw, USA) is widely used to achieve a better fit and fill in both the metaphyseal and diaphyseal medullary canal and can also adjust the excessive anteversion [32–34]. Considering the cylindrical shape of the S-ROM distal stem, we chose the best-fitting circle to evaluate the medullary canal quantitatively instead of ML and AP canal widths. To avoid the periprosthetic femoral fractures, the diameter of fitting circle makes more sense than the longest canal width, which can also eliminate the influence of rotational deformity [4]. In the present study, we found that diameters in both DDH groups were significantly smaller than that in the normal group from C_20_ to C_−40_ and at the level of isthmus. The diameters of IVa femurs were smaller than IVb femurs from C_+20_ to C_−30._ Moreover, the dimensional discrepancies of diameters with the normal group at each level were notably reduced, especially compared with ML canal widths. Theoretically, we believed that the diameter parameter might have important clinical relevance for oriented prosthesis design of the DDH femoral stem.

For depicting the characteristic of the medullary canal of Crowe IV DDH, the proximal femur was divided into metaphyseal, diaphyseal and overall three regions. In the metaphyseal region, all the dimensional indices of IVb were comparable with normal femurs. However, the D_+20_/D_−20_ of IVa group was larger than that of IVb group. In the diaphyseal region, all indices in DDH groups were smaller and IVa femurs were more stovepipe-like than IVb femurs in all the dimensions. Totally, CFIs were smaller in DDH femurs in the ML but not the AP dimension. Further, the index of IVa femur was smaller than IVb only in the ML dimension but comparable in D_+20_/Di. Similarly, Wang et al. [27] also reported that femurs without false acetabulum had a lower CFI in the ML dimension but not the AP dimension. Under the stress of false acetabulum, medullary deformity of IVb femur mainly occurred in the diaphyseal region. By contrast, the medullary deformity occurred in the whole IVa proximal femur.

When performing the femoral reconstruction in THA, intraoperative periprosthetic fracture is proved to be associated with the abnormal morphology of femoral medullary canal [7, 35]. First, all dimensional parameters of Crowe IV femurs were significantly narrower in both metaphyseal and diaphyseal regions. In this regard, the modular femoral stem can offer both regional implant size and effectively segmental fixation. Second, medullary canal of IVa femurs was notably narrower and more dysplastic in the metaphyseal region. As reported [36], most intraoperative periprosthetic femoral fractures were classified as Vancouver Type A and Type B. The application of prophylactic cerclage wire in the metaphyseal region before the proximal cone reaming may effectively avoid relative complications. Last, 3D reconstruction and quantitative analysis in the present study can offer accurate anatomic information of dysplastic femurs. Accordingly, comprehensive preoperative planning is essential for implant prediction and complication avoidance.

The limitations of our study should be noted. First, the sample size was relatively small. However, the Crowe IV hips are uncommon and the results of statistical analysis indicated reliable reproducibility. Further study needs to be done to recruit more high dislocated patients to make the results more conclusive. Second, our study only focused on the morphological features of the proximal femur without results of the corresponding clinical complications and implant survival. Further combined studies will provide more valuable clinical guidance.

## Conclusions

High dislocated femurs are associated with more anteverted femoral neck, smaller femoral offset and narrower medullary canal. Without stimulation of the false acetabulum, IVa femurs were associated with higher dislocation and notably narrower medullary canal, whose variation of medullary canal located in the whole proximal femur. The 3D quantitative analysis in our study may have important clinical relevance for surgeons who perform femoral reconstruction in Crowe type IV hips.

## Data Availability

All the data used and/or analyzed during this study are available upon reasonable request from the corresponding author.

## Abbreviations

DDH: Developmental dysplasia of the hip
THA: Total hip arthroplasty
2/3D: Two-/three-dimensional
FH: Femoral head
GT: Greater trochanter
ML width: Mediolateral (ML) width
AP width: Anterolateral (AP) width
CFI: Canal flare index
MCFI: Metaphyseal canal flare index
DCFI: Diaphyseal canal flare index
